# Air quality index from charcoal production sites, carboxyheamoglobin and lung function among occupationally exposed charcoal workers in South Western Nigeria

**DOI:** 10.1186/s40064-016-3227-9

**Published:** 2016-09-13

**Authors:** O. O. Olujimi, G. R. E. E. Ana, O. O. Ogunseye, V. T. Fabunmi

**Affiliations:** 1 Department of Environmental Management and Toxicology, Federal University of Agriculture, Abeokuta, Ogun State Nigeria; 2 Department of Environmental Health Sciences, Faculty of Public Health, College of Medicine, University of Ibadan, Ibadan, Nigeria

**Keywords:** Gases emission, Air quality index, Lung function, Carboxyheamoglobin, Charcoal production, Nigeria

## Abstract

Charcoal production is often accompanied with gaseous and particulate emission into the atmosphere and occupationally exposed workers could be affected. This cross sectional comparative study was carried out to assess the levels of carbon monoxide (CO), carbon dioxide (CO_2_), sulphur dioxide (SO_2_), nitrogen dioxide (NO_2_) and particulate matter (PM_2.5_) generated during the phases of charcoal production and their relationship with certain biomarkers among charcoal workers (subjects) and non-charcoal workers (controls) such as carboxyhaemoglobin (COHb), forced expiratory volume in the first second of expiration (FEV_1_), peak expiratory flow rate (PEFR) and body mass index (BMI) in Igbo-Ora, Oyo State and Alabata, Ogun State, which are two of the major hubs of charcoal production in South Western Nigeria. Four communities in Igbo-Ora and six communities in Alabata were purposively selected and levels of pollutant gases were assessed using appropriate gas meters, PM_2.5_ was assessed with Thermo Scientific MIE pDR-1500, FEV_1_ and PEFR were measured with Piko-1 spirometer while COHb was assessed using non-invasive pulse CO-oximeter (Rad 57). Data were statistically analyzed and results were compared with recommended guidelines. The mean FEV_1_, PEFR, COHb and BMI for subjects and controls were 2.35 ± 0.73 and 2.69 ± 0.56, 253.72 ± 103.45 and 330.02 ± 94.61 (p < 0.01), 13.28 ± 3.91 and 8.50 ± 3.68 (p < 0.01) and 21.97 ± 2.19 and 23.36 ± 3.74 (p < 0.05) respectively. There was a statistically significant difference between actual and expected values of FEV_1_ (p < 0.01) and PEFR (p < 0.01) among charcoal workers. There existed a positive correlation between CO and COHb while FEV_1_ and PEFR correlated negatively with PM_2.5_. The study showed that charcoal workers are exposed to high levels of CO and PM_2.5_, contributing to lowered respiratory functions for FEV_1_ and PEFR and high levels of COHb compared to the control group. Routine respiratory and carboxyheamoglobin assessment of persons involved in charcoal production is also recommended.

## Background

Emissions from biomass burning is known to generate a large number of air pollutants e.g. respirable particulate matter (PM), carbon monoxide (CO), nitrogen oxides (NOx), sulphur oxides (SOx), formaldehyde, benzene, 1, 3-butadiene, polycyclic aromatic hydrocarbon (PAH) including carcinogens such as benzo[a]pyrene and other toxic organic compounds that can damage human health (Ezzati et al. 2000; Mishra and Retherford 2007). Exposure to air pollutants has been linked with pneumonia, reduced birth weight, acute respiratory infection (ARI) and early mortality in children while adults experience chronic obstructive pulmonary disease (COPD), chronic bronchitis (Bruce et al. 2000; Mishra and Retherford 2007; Fullerton et al. 2009; Po et al. 2011). Other health effects include lung cancer, asthma, cancer of the nasopharynx and larynx, tuberculosis, perinatal conditions, diseases of the eye e.g. cataract and blindness (Bruce et al. 2000; Ezzati and Kammen 2001a, b; Barone-Adesi et al. 2012; Edokpa and Ikelegbe 2012; Kurmi et al. 2012).

There has been major shift from the use of petroleum products and electricity due to high cost and epileptic power supply to the use of charcoal in both the rural and urban centers in Nigeria. This shift have resulted in high demand for charcoal with attendant environmental and health effects. Over 90 % of studies on charcoal production in Nigeria have mainly focused on the economic benefit of the production vis-à-vis poverty alleviation of the rural populace involved in charcoal production and influence of wood species on the properties of charcoal (Ogunsanwo et al. 2007; Tunde et al. 2013; Po et al. 2011). The charcoal industry is a source of earning extra income for a large number of farmers and rural workers who reside in these centers, and, for some individuals, it is the primary source of income they depend on to support their families. The rudimentary process of charcoal production entails carbonization of wood with consequent release of smoke from kiln set ups that charcoal workers are continuously exposed to (Souza et al. 2005).

Charcoal production releases wood smoke that contains a wide variety of pollutants such as PM, PAH, CO, NOx, SOx, volatile organic compounds (VOCs) etc. Particulates can serve as vehicles for the transport of microorganisms such as viruses and bacteria to the lungs and blood stream. These pollutants affect public health as they can contribute to the development of cancer, heart and lung disease and reduce the body’s ability to transport oxygen in the case of CO exposure (Ghosh et al. 1996; Anon 2011). Oxides of nitrogen and sulphur cause lung irritation leading to inflammation of the air passage thereby triggering airway obstruction and other more severe effects (Dost 1991). Pyrolysis, being a critical phase in charcoal production is usually monitored closely by someone who is responsible for the outcome of the process. The activity exposes the person to large amount of inhalable smoke for a period of 3–5 days (Souza et al. 2005). Crystalline silica, one of the major components of particulate matter, has also been classified as a known human carcinogen and is associated with systemic autoimmune disease (IARC 1997).

In a study conducted in Greece among charcoal workers by Tzanakis et al. (2001), it was reported that cough, expectoration, wheezing and dyspnea were significantly more prevalent among charcoal workers when compared to individuals who were not exposed to smoke. The increase in the prevalence of respiratory disorders may be due to genetic mutation which can be triggered by environmental factors, thereby leading to allergic symptoms (Ediagbonya and Tobin 2013). Patients with pre-existing respiratory or heart-related issues are usually at risk of contracting the most severe adverse health effects caused by exposure to inhalable particles (Schwartz and Dockery 1992; Sunyer et al. 1993).

Chronic exposure to particulate matter during childhood or adolescence can greatly reduce lung function (Gauderman 2004). Even acute exposure to wood smoke has been associated with increased risk of respiratory symptoms (Svedahl et al. 2009; Orozco-Levi et al. 2006). Obligatory continuous consumption, which is a unique property of air is demystified in that an average adult only requires about 1.4 kg of food, 2 kg of water while he requires about 14 kg of air each day. This is the rationale for unending research on air pollution (Ediagbonya and Tobin 2013). Lung diseases are basically of two types; obstructive and restrictive (Abuzant et al. 2015). Asthma is an example of obstructive lung disease (Colledge et al. 2010) while idiopathic lung fibrosis is an example of restrictive lung disease (Raghu et al. 2011). The mechanism of action of the former is that it blocks airway insomuch that air movement is impeded while the mechanism of action of the latter is such that there exists insufficient lung expansion (Abuzant et al. 2015). However, some lung diseases exhibit both obstructive and restrictive attributes (Gardner et al. 2011).

Spirometer is widely used in assessing lung function. It measures the volume and flow rate of inspired and expired air. Lung function tests (LFTs) are used to distinguish obstructive and restrictive diseases and determine the degree of associated changes (Colledge et al. 2010; Holguin 2012). Forced Expiratory Volume in the first second (FEV_1_) and Forced Vital Capacity (FVC) are two of the parameters measured by a spirometer (Quadrelli et al. 2007). The peak expiratory flow rate (PEFR) is the maximum flow achieved during an expiration delivered with maximal force starting from the level of maximum lung inflation. Value obtained may vary depending on the properties and preferences of the instrument used (Quanjer et al. 1997). PEFR is important in the routine assessment of healthy and asthmatic children (Seck 1991). FEV_1_ is the maximal volume of air exhaled in the first second of a forced expiration from a position of full inspiration (Miller et al. 2005; Abuzant et al. 2015). FVC is the maximal volume of air exhaled with maximally forced effort from a maximal inspiration. In other words, it is the vital capacity performed with a maximally forced expiratory effort, expressed in litres at body temperature and ambient pressure saturated with water vapour (Miller et al. 2005).

The most important health effects associated with exposure to CO are due to its strong bond with the hemoglobin molecule, forming carboxyhaemoglobin (COHb). The COHb impairs the oxygen-carrying capacity of the blood, putting a strain on tissues with high oxygen demand, such as the heart and the brain. Carbon monoxide also binds to cytochrome oxidase, which could reduce the cells’ ability to utilize oxygen (Ward 1999; WHO 1999; Varon et al. 1999). Studies on emission of pollutant gases and particles from charcoal production activities and associated health status of occupationally exposed charcoal workers in rural areas of Nigeria are scanty. Thus, this study aimed to assess (1) the impact of charcoal production on air quality from pyrolysis and charcoal removal activities and (2) to assess the human respiratory health status among the occupationally exposed workers.

## Methods

### Study areas

This research is based on comparative cross-sectional study. It entails the use of questionnaires to obtain socio-demographic data, monitoring of gases (CO, CO_2_, NO_2_ and SO_2_) and particulate matter (PM_2.5_) with subsequent determination of how the lung functions and carboxyheamoglobin among occupationally exposed charcoal workers and non-charcoal workers. The study was conducted at Igbo-Ora and Alabata settlements in Oyo and Ogun States respectively.

### Study population

The participants selected for this study were charcoal workers and non-charcoal workers in Igbo-Ora and Alabata. Socio-demographic characteristics such as age, gender, educational status, marital status, religion and tribe were obtained from the participants.

#### Inclusion criteria for charcoal workers

Must be engaged in charcoal production within any of the selected settlements.

Must have been involved in charcoal production at least 1 month before the data were collected.

#### Inclusion criteria for non-charcoal production workers

Must not be engaged in charcoal production within any of the selected settlements.

Must not be involved in charcoal production but live within the selected settlements.

#### Sampling procedure

Purposive sampling technique was used to select the communities for this study Alabata in Ogun State and Igbo-ora in Oyo State. These communities were two of the major hubs of charcoal production in South Western Nigeria. Estimated sample size of 298 charcoal workers of the settlements in Alabata and Igbo-Ora were selected systematically while 298 non-charcoal workers were selected based on the inclusion criteria for non-charcoal workers. The sample size for the study was 298 respondents per group (i.e. 298 subjects and 298 controls). Alabata and Igbo-ora, had 149 subjects and 149 respectively. However, the selected communities differ in the number of charcoal workers, hence, proportional allocation was applied. A list containing the names of all charcoal workers in the selected communities was obtained from the secretary of their union and simple arithmetic was used to determine the number of charcoal workers to be selected in each community.

$${\text{P}}.{\text{A}}. = \frac{{{\text{Number}}\,{\text{of}}\,{\text{charcoal}}\,{\text{workers }}\,{\text{in}}\,{\text{each}}\,{\text{community}}}}{{{\text{Total}}\,{\text{number}}\,{\text{of}}\;{\text{charcoal}}\,{\text{workers}}\,{\text{in}}\;{\text{selected}}\,{\text{communities}}}} \times {\text{Sample}}\,{\text{size}}$$

where P.A. = Proportional allocation.

Proportional allocation gives the number of participants to be selected from the list of charcoal workers in each community. The participants for this study were then selected using systematic random sampling where the required numbers of charcoal workers derived from proportional allocation were selected using a sampling interval *K*. The interval *K* was calculated by dividing the number of charcoal workers on the list by value derived from proportional allocation. The sampling was done by selecting a name on list randomly and then every *K*th name was selected until the required numbers of subjects were selected. Hence, the list containing names of charcoal workers in each community served as the sampling frame. The survey was carried out from May to July, 2015. The survey instrument was initially designed in English, translated to local languages, and then translated back to English by a translator to ensure that the translated version captured the questions correctly. The interviews were conducted in Yoruba, Tiv, Igbo and Hausa which were the primary languages of the interviewees. In order to ensure accuracy and validity, the field data collected were recorded on the field data sheets and were double-entered. A semi-structured interviewer administered questionnaire was used to obtain information on the common health hazards of participants associated with charcoal production and non-charcoal workers as controls.

#### Data collection for gases and human exposure assessment

Monitors were placed within 1 m radius of the kiln. The 1 m was used because charcoal workers keep an average distance of 1 m to the kilns (smoke sources) during charcoal production. The aim of the monitoring was to assess the concentration of pollutant gases and particles viz-a-viz exposure by charcoal workers. The charcoal production activities monitored for this study were pyrolysis and removal. Carbon monoxide was monitored using Extech CO 10 m. 4 h monitoring of CO was carried out during pyrolysis with measurements taken at 5 min intervals. The CO meter was calibrated before use and during period of monitoring by zeroing at regular intervals. Carbon dioxide was monitored using Telaire 7001 carbon dioxide and temperature monitor. 4 h monitoring of CO_2_ was carried out during pyrolysis with measurements taken at 5 min intervals. The CO_2_ m was calibrated before use and during period of monitoring by zeroing at regular intervals. Sulphur dioxide was monitored using Z-1300 Sulfur dioxide meter during pyrolysis for 4 h. The measurements were taken at 5 min intervals. The SO_2_ m was calibrated before use and during period of monitoring by zeroing at regular intervals. Nitrogen dioxide (NO_2_) was monitored during pyrolysis for 4 h using Z-1400 Nitrogen dioxide meter and readings were recorded at 5 min interval. The NO_2_ m was calibrated before use and during period of monitoring by zeroing at regular intervals according to the manuals. Particulate matter was monitored using MIE pDR-1500 Active Personal Particulate Monitor. Monitoring of particulate matter (PM_2.5_) was carried out during the pyrolysis and removal processes. The measurements were taken are real time. The equipment were placed about one (1) m from the production stand. However, monitoring during charcoal removal processes varied between 2 and 4 h depending on the size of the earth kiln and the number of people involved. During the removal process, monitoring of CO, CO_2_, SO_2_ and NO_2_, were also done and measurements were taken at 5 min intervals.

Spirometry examination was performed using a calibrated Piko-1 spirometer to assess forced expiratory volume in the first second of expiration (FEV_1_) and peak expiratory flow rate (PEFR) in order to determine the lung function of respondents. Each subject was made to complete a dynamic spirometry with at least three acceptable and two reproducible maneuvers according to standard guidelines. Expected values for FEV_1_ and PEFR were derived via equations reported by Ingle et al. (2005) using age and height of respondents. Non-invasive pulse CO-oximeter (Rad-57) was used to assess the COHb level of research participants. Weighing balance and meter rule were used to take anthropometric measurements of charcoal workers and non-charcoal workers in the study areas from which body mass index (BMI) was calculated.

#### Air quality index (AQI)

Air quality index (AQI) is a standardized method for assessing the quality of air using five criteria pollutants (ground level ozone, SO_2_, NO_2_, CO and PM). Four of them (SO_2_, NO_2_, CO and PM_2.5_) were monitored in this study.

AQI was calculated using the formula below:

$$I_{p} = \frac{{I_{HI} - I_{LO} }}{{BP_{HI} - BP_{LO} }} \left( {C_{P} - BP_{LO} } \right) + I_{LO}$$

Cp = the rounded concentration of pollutant p; Ip: the index for pollutant p; I_HI_: the AQI value corresponding to BP_HI_; I_LO_: the AQI value corresponding to BP_LO_; BP_HI_ = the breakpoint that is greater than or equal to Cp; BP_LO_ = the breakpoint that is less than or equal to Cp (USEPA 2006).

#### Data analysis

Data was entered and analysed using statistical package for the social sciences (SPSS) version 20. Descriptive and inferential statistics were used in this study. Descriptive statistics was used to summarize data. Mean ± standard deviation (SD) and range was calculated for the emissions (CO, CO_2_, SO_2_, NO_2_ and PM_2.5_) and biomarkers (FEV_1_, PEFR COHb and BMI) of respondents. *T* test was used to test for any significant differences in FEV_1_, PEFR, COHb and BMI between respondent groups (subject and controls). *T* test was also used to compare actual and expected values of FEV_1_ and PEFR. Simple linear regression was used to determine the relationship between two quantitative variables. Pearson correlation test was carried out to check for relationships between quantitative variables. Smoking status of respondents was determined before recruitment for the study. Only non-smokers were selected to reduce the number of potential confounding variables. Multiple linear regression was used to statistically test for the confounding effect of the use of biomass fuel for household cooking. Use of biomass fuel was not a statistically significant predictor of the carboxyheamoglobin (COHb) and lung function (PEFR and FEV1).

## Results

### Socio-demographic characteristics of respondents

**Fig. 1:**
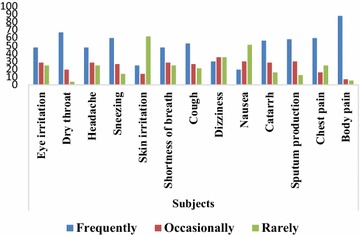
Perception of charcoal workers to common symptoms and conditions

Table 1 shows the socio-demographic characteristics of respondents. Majority of charcoal workers were within age 21–30 years (43.9 %), males (73.7 %), married (73.7 %), Christians (86.0 %), Tiv (64.9 %) with no education (35.1 %). The mean age of charcoal workers was 32.67 ± 10.47. Majority of non-charcoal workers were within age 31–40 years (38.6 %), males (71.9 %), married (56.1 %), Christians (63.2 %), Yoruba (66.7 %) with tertiary education (36.8 %). The mean age of non-charcoal workers was 35.46 ± 12.82 (Fig. 1).

**Table 1:** Socio-demographic characteristics of respondents

Socio-demographic characteristics	Subgroups	Percentage	
		Subjects	Controls
Mean age of subjects 32.67 ± 10.47 Mean age of controls 35.46 ± 12.82	≤20	10.5	7.0
	21–30	43.9	35.1
	31–40	31.6	38.6
	41–50	7.0	5.3
	>50	7.0	14.0
Gender	Male	73.7	71.9
	Female	26.3	28.1
Educational status	No education	35.1	17.5
	Primary education	31.6	21.1
	Secondary education	31.5	24.6
	Tertiary education	1.8	36.8
Marital status	Married	73.7	56.1
	Single	26.3	43.9
Religion	Christianity	86.0	63.2
	Islam	14.0	36.8
Tribe	Yoruba	12.3	66.7
	Tiv	64.9	21.1
	Hausa	–	12.2
	Others	22.8	–

### Perception of respondents to common symptoms and conditions

**Fig. 2:**
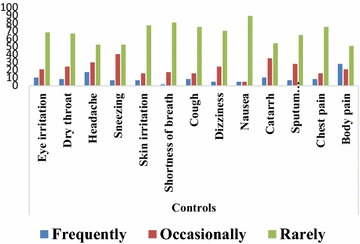
Perception of non-charcoal workers to common symptoms and conditions

**Fig. 3:**
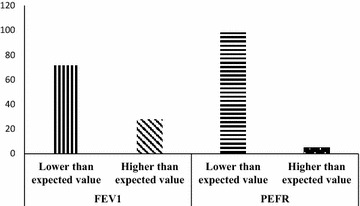
Proportion of charcoal workers with FEV1 and PEFR values lower or higher than expected values

Figures 2 and 3 showed the perception of charcoal workers and non-charcoal workers to common symptoms and conditions. Majority of charcoal workers experienced eye irritation (47.4 %), dry throat (66.7 %), headache (47.4 %), sneezing (59.6 %), shortness of breath (47.4 %), cough (52.6 %), catarrh (56.1 %), sputum production (57.9 %), chest pain (59.6 %) and frequent body pain (87.7 %) whereas majority of non-charcoal workers experienced eye irritation (68.4 %), dry throat (66.7 %), headache (52.6 %), sneezing (52.6 %), skin irritation (77.2 %), shortness of breath (80.7 %), cough (75.4 %), dizziness (70.2 %), nausea (89.4 %), catarrh (54.4 %), sputum production (64.9 %), chest pain (75.4 %) and occasional body pain (50.9 %).

### Emissions from pyrolysis, removal processes and air quality index

The mean concentration of the gases and PM_2.5_ monitored at the study areas are presented in Tables 2 and 3. At Alabata settlements during the pyrolysis process, NO_2_ varied from 0.19 ± 0.22 ppm (Ikugba) to 0.48 ± 0.71 ppm (Ayogun); SO_2_ varied from 1.74 ± 2.19 ppm (Raka) to 2.93 ± 3.21 ppm (Ayogun); CO ranged from 2.93 ± 3.21 ppm (Ayogun) to 585 ± 163 ppm (Ikugba); CO_2_ varied from 317 ± 147 ppm (Raka) to 3877 ± 2608 ppm (Ikugba) while PM_2.5_ varied from 103 ± 191 µgm^−3^ (Fojubaye) to 34,000 ± 17,000 µgm^−3^ (Ikugba). At Igbo-Ora settlements, NO_2_ varied from 0.69 ± 0.74 (Apata) to 1.85 ± 1.53 ppm (Iyana babanla); SO_2_ varied from 0.8 ± 0.51 ppm (Iyana babanla) to 3.82 ± 2.48 ppm (Igboyange); CO ranged from 408 ± 116 ppm (Apata) to 959 ± 240 ppm (Irepodun); CO_2_ varied from 2045 ± 132 ppm (Apata) to 3281 ± 1952 ppm (Igboyange) and PM_2.5_ varied from 44,000 ± 17,000 µgm^−3^ (Igboyange) to 328,000 ± 87,000 µgm^−3^ (Irepodun). Similar trend was obtained for the gases and PM_2.5_ monitored during the removal process at all the sites. Additionally, it is worthy to note that the concentration of all the gases and PM_2.5_ monitored were significantly (p < 0.05) higher at Igbo-Ora compared to Alabata. This thus, shows that there are more charcoal production activities in Igbo-Ora area than in Alabata area. The air quality indexes of the settlements are presented in Table 4. All the settlements investigated recorded poor air quality indexes except the Adaako area that recorded good air quality index for CO and SO_2_ and Fojubaye with good air quality index for SO_2_.

**Table 2:** Range and mean ± SD of pyrolysis and charcoal harvesting at settlements in Alabata, Ogun State

	Alabata (pyrolysis)				Alabata (harvesting)		
	Oluwotiti	Raka	Ikugba	Ayogun	Adaako	Raka	Fojubaye
NO_2_ (ppm) mean ± SD	0.25 ± 0.47	0.20 ± 0.27	0.19 ± 0.22	0.48 ± 0.71	0.018 ± 0.025	0.031 ± 0.058	0.08 ± 1.35
SO_2_ (ppm) mean ± SD	1.89 ± 2.77	1.74 ± 2.19	2.85 ± 1.83	2.93 ± 3.21	0.0075 ± 0.011	0.012 ± 0.016	0.02 ± 0.05
CO (ppm) mean ± SD	280 ± 149	96.8 ± 57.1	585 ± 163	2.93 ± 3.21	1.50 ± 2.05	14.65 ± 5.31	13.4 ± 6.46
CO_2_ (ppm) mean ± SD	1986 ± 1648	964 ± 819	3877 ± 2608	3021 ± 1958	221 ± 27	317 ± 147	714 ± 673
PM_2.5_ (µg/m^3^) mean ± SD	17,000 ± 270,000	6550 ± 7520	34,000 ± 17,000	18,000 ± 9000	430 ± 110	279 ± 230	103 ± 191

**Table 3:** Concentration of gases and particulate matter from pyrolysis for settlements in Igboora, Oyo State

	Igbo-Ora (pyrolysis)				Igbo-Ora (harvesting)			
	Igboyange	Apata	Irepodun	Iyana babanla	Igboyange	Apata	Irepodun	Iyana babanla
NO_2_ (ppm) mean ± SD	1.30 ± 1.53	0.69 ± 0.74	1.67 ± 1.34	1.85 ± 1.53	0.097 ± 0.04	0.39 ± 0.40	0.35 ± 0.46	0.15 ± 0.14
SO_2_ (ppm) mean ± SD	3.82 ± 2.48	0.87 ± 0.83	2.93 ± 3.06	0.80 ± 0.51	0.00	0.08 ± 0.21	0.04 ± 0.06	0.05 ± 0.12
CO (ppm) mean ± SD	691 ± 111	408 ± 116	959 ± 240	492 ± 202	1.83 ± 1.20	64.3 ± 67.5	32.7 ± 45.8	80 ± 102
CO_2_ (ppm) mean ± SD	3281 ± 1952	2045 ± 132	2952 ± 1813	2863 ± 1547	236.89 ± 15.57	517 ± 169	584 ± 183	1222 ± 1355
PM_2.5_ (µg/m^3^) mean ± SD	44,000 ± 17,000	50,600 ± 22,800	328,000 ± 87,000	70,400 ± 15,400	540 ± 210	450 ± 210	480 ± 220	540 ± 250

**Table 4:** Air quality index of study sites

Location	AQI (SO_2_)	AQI (NO_2_)	AQI (CO)	PM_2.5_
Oluwotiti, Alabata	Extremely hazardous	Not applicable	Extremely hazardous	Extremely hazardous
Adaako, Alabata	7.8 (good)	Not applicable	12.4 (good)	356.4 (hazardous)
Raka, Alabata	Extremely hazardous	Not applicable	Extremely hazardous	Extremely hazardous
Ikugba, Alabata	Extremely hazardous	Not applicable	Extremely hazardous	Extremely hazardous
Ayogun, Alabata	Extremely hazardous	Not applicable	Extremely hazardous	Extremely hazardous
Fojubaye, Alabata	25 (good)	Not applicable	166.2 (unhealthy)	172.8 (unhealthy)
Igboyange, Igboora	Extremely hazardous	Not applicable	Extremely hazardous	Extremely hazardous
Apata, Igboora	343.3 hazardous	Not applicable	Extremely hazardous	Extremely hazardous
Irepodun, Igboora	Extremely hazardous	331.5 (hazardous)	Extremely hazardous	Extremely hazardous
Iyana Babanla, Igboora	298.7 (very unhealthy)	334.0 (hazardous)	Extremely hazardous	Extremely hazardous

### Biomarker assessment of respondents and relationships with emissions during charcoal production

The mean and standard deviation (SD) of FEV_1_, PEFR, COHb and BMI among charcoal workers and non-charcoal workers are presented in Table 5. The FEV_1_ for charcoal and non-charcoal workers were 2.35 ± 0.73 and 2.69 ± 0.56 respectively while the PEFR for charcoal workers and non-charcoal workers were 253.72 ± 103.45 and 330.02 ± 94.61 respectively (p < 0.01). The COHb for charcoal workers and non-charcoal workers were 13.28 ± 3.91 and 8.50 ± 3.68 respectively (p < 0.01) while the BMI for charcoal and non-charcoal workers were 21.97 ± 2.19 and 23.36 ± 3.74 respectively (p < 0.05). The comparison between actual and expected mean values for FEV_1_ and PEFR among charcoal workers is presented in Table 6. There was a statistically significant (p < 0.01) difference between actual and expected values of FEV_1_ and PEFR among charcoal workers. Figure 3 shows the proportion of charcoal workers with FEV_1_ and PEFR values lower or higher than expected values. The results showed that majority of charcoal workers recorded FEV_1_ (71.9 %) and PEFR (98.2 %) values lower than the expected values.

**Table 5:** Comparison of biomarkers between respondent types

	Subject	Control	p-value
FEV_1_			
Range	0.88–4.21	1.49–4.02	
Mean ± SD	2.35 ± 0.73	2.69 ± 0.56	0.06
PEFR			
Range	104.00–618.00	156.00–650	
Mean ± SD	253.72 ± 103.45	330.02 ± 94.61	0.000
COHb (%)			
Range	5.00–20.00	1.00–18.00	
Mean ± SD	13.28 ± 3.91	8.50 ± 3.68	0.000
BMI (kg/m^2^)			
Range	16.22–26.03	18.03–35.06	
Mean ± SD	21.97 ± 2.19	23.36 ± 3.74	0.019

**Table 6:** Comparison of actual and expected values of FEV_1_ and PEFR

	FEV1 (actual)	FEV1 (expected)	p value
Subjects	2.35 ± 0.73	2.82 ± 0.40	0.000

	PEFR (actual)	PEFR (expected)	
Subjects	253.72 ± 103.45	529.61 ± 41.52	0.000

**Fig. 4:**
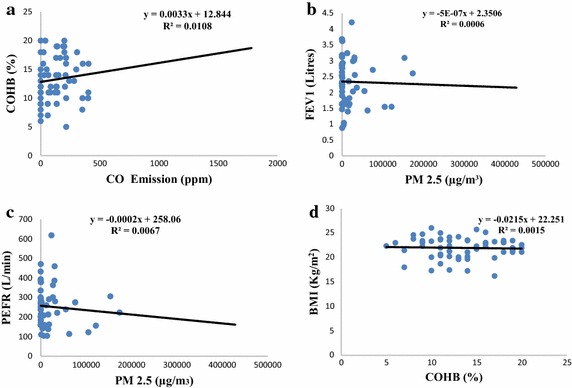
**a** Correlation between CO and COHB of workers. **b** Linear relationship between PM_2.5_ and FEV1. **c** Linear relationship between PM_2.5_ and PEFR. **d** Linear relationship between BMI and COHB

The relationships between emissions during pyrolysis and biomarkers assessed among charcoal workers are presented in Table 7. Nitrogen dioxide correlated positively with COHb (r = 0.074) and FEV_1_ (r = 0.036) while it correlated negatively with PEFR (r = −0.017) and BMI (r = −0.071). Sulphur dioxide correlated negatively with COHb (r = −0.169), FEV_1_ (r = −0.144) and PEFR (r = −0.14) while it correlated positively with BMI (r = 0.061). The value of CO showed negative correlation with FEV_1_ (r = −0.173), PEFR (r = −0.077) and BMI (r = −0.116) while it correlated positively with COHb (r = 0.104). The strength of the linear relationship between the levels of CO and COHb (R^2^ = 1.08 %) is depicted in Fig. 4. The CO_2_ correlated positively with COHb (r = 0.038) and PEFR (r = 0.024) while it correlated negatively with FEV_1_ (r = −0.005) and BMI (r = −0.010). There existed a significant positive correlation between PM_2.5_ and COHb (r = 0.320, p < 0.05), while PM_2.5_ correlated negatively with FEV_1_ (r = −0.027), PEFR (r = −0.082) and BMI (r = −0.144). Furthermore, BMI correlated positively with FEV_1_ (r = 0.328, p < 0.05) and PEFR (r = 0.279, p < 0.05) while it correlated negatively with COHb (r = −0.038). The strength of the linear relationship PM_2.5_ recorded with FEV_1_ (R^2^ = 0.06 %) and PEFR (R^2^ = 0.67 %) are shown in Fig. 4.

**Table 7:** Correlation between the quantitative variables

Pearson correlation coefficient Sig (2 tailed)	NO_2_	SO_2_	CO	CO_2_	PM_2.5_	COHb	FEV_1_	PEFR	Body mass index
NO_2_	1								
SO_2_	0.181** 0.000	1							
CO	0.477** 0.000	0.390** 0.000	1						
CO_2_	0.261** 0.000	0.422** 0.000	0.525** 0.000	1					
PM	0.441** 0.000	0.212** 0.000	0.717** 0.000	0.261** 0.000	1				
COHb	0.074 0.586	−0.169 0.209	0.104 0.441	0.038 0.781	0.320* 0.015	1			
FEV_1_	0.036 0.789	−0.144 0.286	−0.173 0.199	−0.005 0.973	−0.027 0.843	−0.075 0.577	1		
PEFR	−0.017 0.902	−0.140 0.300	−0.077 0.571	0.024 0.859	−0.082 0.545	−0.122 0.367	0.875** 0.000	1	
BMI	−0.071 0.599	0.061 0.655	−0.116 0.392	−0.010 0.943	−0.144 0.284	−0.038 0.777	0.328* 0.013	0.279* 0.036	1

* Correlation is significant at the 0.05 level (2-tailed); ** correlation is significant at the 0.01 level (2-tailed)

## Discussion

Majority of charcoal workers experienced most of the reported symptoms and conditions such as eye irritation, dry throat, headache, sneezing, shortness of breath, cough, catarrh, sputum production, chest pain and frequent body pain whereas non-charcoal workers rarely experienced these symptoms and conditions. This may be due to emissions arising from charcoal production and adverse working conditions these charcoal workers are exposed to during and after the phases of charcoal production. This assertion is supported by Souza et al. (2005) who stated that work environment has influence on human health. In a study (Ediagbonya and Tobin (2013) that assessed air pollution and respiratory morbidity among 400 respondents in Sapele, Nigeria, shows the prevalence of respiratory symptoms such as cough (10.5 %), phlegm (21.3 %), wheezing (13.5 %), difficulty in breathing (14.8 %), chest pain (13.8 %) and sore throat (10.3 %).

Cough alongside other symptoms such as sneezing, nasal secretion and sputum production have also been reported as common symptoms in previous studies by Tzanakis et al. (2001), Ibhazehiebo et al. (2007), Swiston et al. (2008), Souza et al. (2005), Keraka et al. (2013) and Adewole et al. (2013). As documented by Dost (1991), increase in cough and sputum expectoration could be explained by stating that aldehydes and acids reduce the ciliary activity of the respiratory tract, thereby interfering with the ability of the airway epithelium to clear mucus and remove particles and micro-organisms. In this study, eye irritation was one of the frequently experienced symptoms among charcoal workers and this may be due to causation of metabolite-induced opacification of the eye lenses by wood smoke which could result in cataract formation and eventually blindness (Rao 1995).

Although, no study to the best of our knowledge had reported gaseous emission from wood pyrolysis during charcoal production activities in Nigeria, previous studies have reported various anthropogenic emissions with varying concentrations of CO, CO_2_, NO_2_, SO_2_ and particulate matter. For example, Akande et al. (2013) reported 20.09 ppm; 0.004 and 0.002 ppm for CO_2_, CO and SO_2_ respectively at marine clay processing plant. Also, Adoki (2012) reported concentration range of 92–430 µgm^−3^ (0.035–0.16 ppm) for SO_2_, and 81.0–150 µgm^−3^ (0.043–0.080 ppm) for NO_2_ in Niger/Delta. Recently, the National Environmental Standards and Regulatory Enforcement Agency in a study conducted in Abuja reported 0.375 ppm; 0.165 ppm; 0.260 and 0.345 ppm for SO_2_, NO_2_, NO and NO_x_. The mean concentration of SO_2_ for the two study locations (Tables 2 and 3) clearly indicated that pyrolysis is a factor in air pollution compared to the removal of charcoal process. The general mean concentration of SO_2_ is above the 0.5 ppm USEPA 3-h permissible limit. However, the reported ranges for charcoal removal process is within the 500 µgm^−3^ (0.19 ppm) WHO 10 min exposure average (WHO 2006). Similarly, NO_2_ concentration reported for all the pyrolysis sites were above the WHO 1-h average time standard of 0.11 ppm.

As presented in Tables 2 and 3, the CO concentration during pyrolysis and harvesting ranged from 2.93 ± 3.21 ppm (Ayogun, Alabata) to 959 ± 240 ppm (Irepodun, Igbo-Ora) and 1.5 ± 2.05 ppm (Adaako, Alabata) to 80 ± 102 ppm (Iyana Babanla. Igbo-Ora). These results indicate that the average CO concentration is above the Nigeria Ambient Air Quality Standard (NAAQS) which stipulates an average concentration of 10–20 ppm for an 8-hourly average time (FEPA 1999). However, except for Apata, Irepodun and Iyana Babanla areas, charcoal removal releases low levels of CO into the atmosphere. The concentration of CO_2_ varied from 964 ± 819 ppm (Raka, Alabata) to 3877 ± 2668 ppm (Ikugba, Alabata) and from 221 ± 27 ppm (Adaako, Alabata) to 1222 ± 1355 ppm (Iyana Babanla, Igbo-Ora) for pyrolysis and removal processes respectively. The concentration for all the settlements was above the 600 ppm maximum natural concentration standard and the recommended WHO threshold limit value of 500 ppm that is safe for adult for an 8-hourly work day.

In addition, PM_2.5_ concentration was generally above the WHO guideline of 25 µgm^−3^ for 24 h mean period. The observed concentration of PM_2.5_ is a pointer towards health effects for the occupationally exposed workers many of which do not make use of personal protective equipment. Also, particulate matter can easily react with polycyclic aromatic hydrocarbons to form photochemical smog in the presence of ultraviolent light. Except for SO_2_ in Adaako and Fojubaye and CO in Adaako, the air quality indexes ranged from unhealthy to extremely hazardous (Table 4).

There was a statistically significant (p < 0.01) difference in PEFR between charcoal and non-charcoal workers. This shows that the reduction in PEFR values among charcoal workers is very evident. Exposure to smoke has implications for significant reduction in mean PEFR values for charcoal workers (253.72 ± 103.45) who were exposed to smoke from kilns used for charcoal production as compared to mean PEFR value for non-charcoal workers (330.02 ± 94.61). This result confirms the studies by Ediagbonya and Tobin (2013) and Ibhazehiebo et al. (2007) that reported reduced PEFR values among subjects exposed to smoke as compared to controls. Several other studies have also reported reduction in PEFR values and increased respiratory symptoms (Alakija et al. 1990; Ellegard 1994; Tzanakis et al. 2001). There was reduction in FEV_1_ of charcoal workers compared to non-charcoal workers which was not significantly. In a study that investigated respiratory symptoms in charcoal production workers in Brazil by Souza et al. (2005) reported that mean FEV_1_ of 65 charcoal production workers was 3.24 ± 0.82 L (93.2 ± 16.0 % of predicted). The FEV_1_ value is higher than that recorded in this study (2.35 ± 0.73). Also, a study conducted in Palestine among workers in charcoal factories, reported that more subjects recorded decreased FEV_1_ values than members of the control group (p = 0.015) Abuzant et al. (2015).

Furthermore, PM_2.5_ correlated negatively with FEV_1_ (r = −0.027) and PEFR (r = −0.082). This result is consistent with the mechanism of action of inhalable particulate matter as lung function decreases with increase in levels of particulate matter especially those with sizes less than 10 microns. The result also showed that there was a statistically significant (p < 0.01) difference in the actual and expected values of FEV_1_ and PEFR among charcoal workers where 71.9 and 98.2 % of charcoal workers recorded FEV_1_ and PEFR values lower than the expected values respectively. This is an indication that charcoal production has a measurable effect on the respiratory health of charcoal workers due to exposure of charcoal workers to high levels of PM_2.5_, thereby reducing lung function assessed through FEV_1_ and PEFR. In addition, components of wood smoke such as SO_2_, NO_2_ and particulate matter have been reported to have adverse effect on the lung function and increase respiratory symptoms even in low concentrations (Gong 1992). The effect of charcoal dusts on lung function can be explained in terms of occupational exposures to particles in which the entrance of dust particles trigger inflammatory reactions, leading to lung fibrosis with attendant reduction in FVC and FEV_1_ values (Longo et al. 2011).

The result of COHb presented in Table 5 showed the range of COHb in charcoal workers is 5–20 % with mean concentration of 13.28 ± 3.91 % while it ranged from 1.00 to 18.00 % with mean concentration of 8.50 ± 3.68 % among controls. Generally, the mean concentration recorded in subjects and controls exceeded the acceptable limit of 2.5 % (WHO 1999). The COHb level of 3–8 % has been reported in regular workers and higher concentration in heavy smokers and drivers working in high traffic density areas (Sen et al. 2010). The result of the study confirms the reported ranges of COHb for subjects and controls. In acute and chronic CO toxicity, the common symptoms includes fatigue, signs of upper respiratory tract infections, dyspnea, chest pain, palpitations, lethargy, confusion, depression, hallucinations, agitation, vomiting, diarrhea, abdominal pain, headache, dizziness, blurred vision, syncope, seizure, urinary incontinence, memory and gait disturbance, neurological disorders, cognitive functions impairment and gradually developing psychiatric symptoms (Van Meter 2010; Nelson and Hoffman 2010; Shochat and Luchessi 2013). Death from acute CO poisoning is not uncommon in developed countries most especially in fire related poisoning (Van Meter 2010). However, several cases of acute death arising from acute CO poisoning have been reported in Nigeria due to exposure to generators.

Levels of COHb in charcoal workers were significantly (p < 0.01) higher than the non-charcoal workers and WHO guideline of 2.5 % (WHO 1999). The mean % COHb among charcoal workers was fivefold higher than the WHO guideline. This indicates that charcoal workers are exposed high levels of CO during charcoal production. This further explains the fact that there was a positive correlation between CO and COHb (r = 0.104, R^2^ = 1.08 %). This is also consistent as COHb levels rise in charcoal workers with increase in CO emission from kiln set ups. High levels of CO resulting in COHb level between 5 and 9 % has been reported in persons exposed to wood smoke (Ellegard 1994). Hoek et al. (2002) reported that average BMI was lower for those exposed to traffic-related air pollutants and higher BMI for those with air pollutants exposure. Significant difference was recorded for BMI between those living near a road and those that do not live near a major road. Similarly, McConnell et al. (2014) reported that exposure to secondhand smoke (SHS), maternal smoking during pregnancy and vehicular air pollution have associations with BMI. Though the mean BMI of charcoal workers and non-charcoal workers fall within normal or healthy weight (NOO 2009), charcoal workers recorded a significantly (p < 0.05) lower BMI than non-charcoal workers. This indicates that charcoal workers have less body mass when compared to non-charcoal workers. The mean BMI reported by Souza et al. (2005) (25.7 ± 3.85 kg/m^2^) is also higher than that recorded in this study (21.97 ± 2.19) for charcoal workers.

## Conclusion and recommendations

This study assessed biomarkers such as FEV_1_, PEFR, COHb and BMI and their relationships with emissions from charcoal production among charcoal workers and non-charcoal workers in Igbo-Ora in Oyo State and Alabata in Ogun State, Nigeria. Majority of charcoal workers experienced frequently most of the symptoms and conditions while non-charcoal workers rarely experienced symptoms. Lung function test carried out on respondents revealed that there was reduction in PEFR and FEV_1_ values among charcoal workers when compared to non-charcoal workers. Levels of COHb among charcoal workers was also significantly higher than that of non-charcoal workers. The mean BMI among charcoal workers was lower than the record for non-charcoal workers. The study also revealed significant relationships between emissions from charcoal production and biomarkers of charcoal workers. This study suggest that charcoal workers should be enlightened on the adverse health effects of emissions arising from charcoal production and trained on ways to reduce their exposures to these emissions. A routine respiratory and carboxyhaemoglobin assessment of persons involved in charcoal production is also recommended.
